# NHS national data opt-outs: trends and potential consequences for health data research

**DOI:** 10.3399/BJGPO.2024.0020

**Published:** 2024-07-10

**Authors:** John Tazare, Alasdair D Henderson, Jessica Morley, Helen A Blake, Helen I McDonald, Elizabeth J Williamson, Helen Strongman

**Affiliations:** 1 Faculty of Epidemiology and Population Health, London School of Hygiene and Tropical Medicine, London, United Kingdom; 2 Oxford Internet Institute, University of Oxford, Oxford, United Kingdom; 3 Faculty of Public Health and Policy, London School of Hygiene and Tropical Medicine, London, United Kingdom; 4 Department of Applied Health Research, Institute of Epidemiology and Health Care, University College London, London, United Kingdom; 5 Department of Life Sciences, University of Bath, Bath, United Kingdom; 6 Department of Infectious Disease Epidemiology (International Health), London School of Hygiene and Tropical Medicine, London, United Kingdom

**Keywords:** epidemiology, large database research, database, primary health care

## Abstract

**Background:**

The English NHS data opt-out allows people to prevent use of their health data for purposes other than direct care. In 2021, the number of opt-outs increased in response to government-led proposals to create a centralised pseudonymised primary care record database.

**Aim:**

To describe the potential impact of NHS national data opt-outs in 2021 on health data research.

**Design & setting:**

We conducted a descriptive analysis of opt-outs using publicly available data and the potential consequences on research are discussed.

**Method:**

Trends in opt-outs in England were described by age, sex, and region. Using a hypothetical study, we explored statistical and epidemiological implications of opt-outs.

**Results:**

During the lead up to a key government-led deadline for registering opt-outs (from 31 May 2021–30 June 2021), 1 339 862 national data opt-outs were recorded; increasing the percentage of opt-outs in England from 2.77% to 4.97% of the population. Among females, percentage opt-outs increased by 83% (from 3.02% to 5.53%) compared with 76% in males (from 2.51% to 4.41%). Across age groups, the highest relative increase was among people aged 40–49 years, which rose from 2.89% to 6.04%. Considerable geographical variation was not clearly related to deprivation. Key research consequences of opt-outs include reductions in sample size and unpredictable distortion of observed measures of the frequency of health events or associations between these events.

**Conclusion:**

Opt-out rates varied by age, sex, and place. The impact of this and variation by other characteristics on research is not quantifiable. Potential effects of opt-outs on research and consequences for health policies based on this research must be considered when creating future opt-out solutions.

## How this fits in

Stark differences in opt-out proportions by geographic area were already present in 2017, following Care.data. We have demonstrated that the number of people opting out of the use of their medical data for uses other than direct care sharply increased following the launch of the General Practice Data for Planning and Research (GPDPR) initiative and varied by key demographic characteristics and region. Opt-outs can impact research using these data in a way that is not possible to mitigate using the data available to researchers. This should be carefully considered when planning future national research databases.

## Introduction

The NHS is the umbrella term for publicly funded healthcare systems in each country of the UK, providing medical services and treatment to residents free at the point of use. It covers a wide range of health services, including GPs, hospitals, and other healthcare facilities. The NHS has collected detailed clinical data to support patient care and inform service development since the 1980s.^1^ Government initiatives recognise the unparalleled potential of these data for research that improves medical knowledge and the health of future patients.^2^ For example, by informing the development and evaluation of effective and cost-efficient NHS services, and the assessment of drug safety and efficacy.^3–5^ Use of these clinical data is governed through legally binding ethical and information governance principles and processes.^6^ These processes aim to build and maintain public trust in the use of patient data by balancing public health benefits while minimising risk of harm to NHS patients; for example, through the disclosure of people’s health status and inaccurate or potentially stigmatising research.^6^ Mechanisms to increase public trust include giving patients the option to 'opt-out', preventing use of the individual’s confidential data for research and planning. Since 25 May 2018, following the failure of Care.data (a planned NHS England national database) owing to problems with public trust in decisions related to data sharing, there has been a single mechanism for national data opt-out in England.^7^ This national data opt-out is applied to most forms of planning and research, including highly regulated de-identified datasets used to improve public health.

On 12 May 2021,^8^ the UK government launched the General Practice Data for Planning and Research (GPDPR) initiative, which planned to create a centralised pseudonymised database comprising of the coded primary care records of patients in England.^9^ Widely shared concerns surrounding the openness and transparency of the proposed GPDPR initiative again led to campaigns advocating people to use the national data opt-out service to prevent their data from being shared.^10,11^ These campaigns and concerns resulted in a large spike in national data opt-outs.^7^

High levels of opt-outs have the potential to negatively impact the ability of researchers to use these data to answer important questions through multiple mechanisms.^12–15^ These problems, and their implications for the collection and sharing of health data, have not previously been clearly quantified or described, partly because once an individual has opted out, their data is no longer available to researchers who wish to study these mechanisms and their impact. We use limited aggregated data provided by the NHS to describe how opt-outs vary by key demographic characteristics over time in the context of the GDPDR launch. We combine this information with hypothetical examples that reflect real-world complexity to discuss the implications of opt-outs for planning and research.

## Method

Our descriptive study uses NHS data from the national data opt-out open data dashboard and publication (2021),^16^ and publicly available data on postcode-level 2019 deprivation data from the UK government.^17,18^ National opt-out data provided the weekly total number and proportion (%) of unique NHS numbers associated with a national opt-out in England over time stratified by each of the following variables: age, sex, region (classified at the Office for National Statistics region [*n* = 7] and former clinical commissioning group [CCG] level [*n* = 107]),^19^ and general practice postcode. We linked general practice-level opt-outs to area-based deprivation data to measure the number and percentage of opt-outs by Index of Multiple Deprivation (IMD) rank; this was achieved by mapping postcode-level IMD information to the corresponding general practice postcode. IMD is a composite score capturing small-area socioeconomic information relating to factors including disability, education, employment, and income.

We calculated the increase in total opt-outs between the end of May 2021 and end of June 2021, and the percentage change in the opt-out proportion over this period, in England and for each age, sex, and region. Additionally, we calculated the weekly opt-out proportion; the count of weekly opt-outs divided by the population who had not yet opted out.^17,18^

We described the shape of the association between total opt-out percentage and IMD rank at the end of June 2021 using a local regression (LOESS) curve, which is a smoothing method that does not require *a priori* specification of a model.^20^ Numbers of units with missing data in each analysis are described in the text, tables, and figures.

All of the data and code used in this study are available at the following GitHub repository: https://github.com/johntaz/nhs-opt-outs.

We considered statistical issues related to sample size and bias, and epidemiological issues related to bias in observational studies of associations to discuss the implications of opt-outs for planning and research. These arise from wider and more complex differences between people who opt-out and remain in the data that we are unable to describe using the demographic data that is available. We therefore describe the theory behind these issues and illustrate the impacts through the use of hypothetical clinical examples that further characterise the complexity of real-world scenarios.

## Results

### Descriptive summary of opt-outs

**Figure 1.**
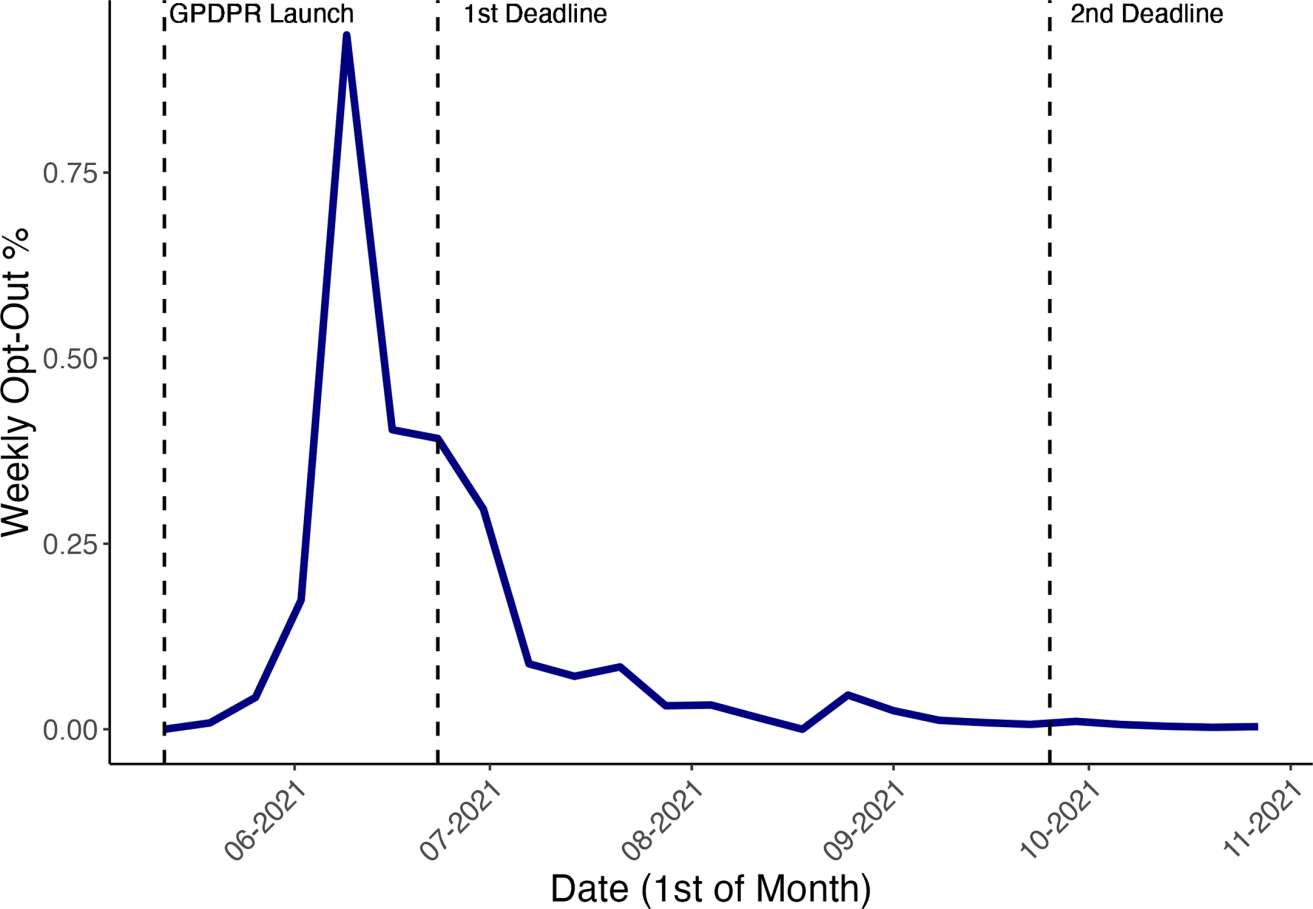
Overall trends in weekly opt-out percentage (blue line) around the national data opt-out deadline (23 June 2021). ‘First’ and ‘second deadline’ refer to national data opt-out deadlines. ‘GPDPR launch’ refers to the launch of the General Practice Data for Planning and Research (GPDPR) initiative for national data opt-outs. Weekly opt-out percentage is defined as weekly opt-out count divided by the population who had not opted out before this time

Figure 1 highlights the sharp increase in the overall percentage of opt-outs during the period leading up to the first opt-out deadline on 23 June 2021. Across the month from the end of May 2021 to the end of June 2021, 1 339 862 national data opt-outs were recorded; increasing the overall percentage of opt-outs from 2.77% (1 683 420/60 798 847)–4.97% (3 023 282/60 860 759) of the population registered with a GP practice in England (Table 1). Figure 1 presents opt-out data until the 1 November 2021, when the final overall percentage of opt-outs was 5.39%.

**Table 1.** Demographics and summaries of national data opt-outs comparing the end of May 2021 and end of June 2021

		End of May 2021			End of June 2021			Summary	
Group		Total opt-outs	Population size	Opt-out %	Total opt-outs	Population size	Opt-out %	Increase in total opt-outs	Per cent change in opt-out %
Overall	–	1 683 420	60 798 847	2.77	3 023 282	60 860 759	4.97	1 339 862	79.41
Age	0–9	83 114	6 639 282	1.25	91 108	6 625 326	1.38	7994	9.85
	10–19	128 132	6 925 392	1.85	196 067	6 933 364	2.83	67 935	52.84
	20–29	229 768	7 968 523	2.88	454 031	7 967 372	5.70	224 263	97.63
	30–39	283 285	8 979 694	3.15	573 671	9 001 495	6.37	290 386	102.02
	40–49	230 275	7 968 107	2.89	481 915	7 976 890	6.04	251 640	109.05
	50–59	229 674	8 210 790	2.80	477 856	8 216 759	5.82	248 182	107.91
	60–69	200 564	6 301 724	3.18	364 238	6 312 974	5.77	163 674	81.28
	70–79	200 111	4 957 245	4.04	272 281	4 970 652	5.48	72 170	35.70
	80–89	85 546	2 337 474	3.66	97 820	2 341 488	4.18	12 274	14.15
	≥90	12 937	510 616	2.53	14 226	514 439	2.77	1289	9.15
	Unknown	14	–	–	69	–	–	–	–
Sex	Female	918 441	30 363 953	3.02	1 679 973	30 389 789	5.53	761 532	82.76
	Male	764 886	30 434 894	2.51	1 343 048	30 470 970	4.41	578 162	75.38
	Unknown	93	–	–	261	–	–	–	–

Opt-out data stratified by age and sex are presented in Supplementary Figure S1 with full data provided in Table 1. This highlights consistently large increases and total percentages of opt-outs, with the highest relative increase among people aged 40–49 years (109%, from 2.89% to 6.04%). By the end of June 2021, people aged 30–39 years had the highest percentage of total opt-outs (6.37%). Total opt-outs by the end of June 2021 were substantially lower in younger children and older adults (0–9 years: 1.38%; ≥90 years: 2.77%).

The percentage of opt-outs was consistently highest in females increasing by 83% (from 3.02% to 5.53%) compared with an increase of 76% in males (2.51% to 4.41%).

**Figure 2.**
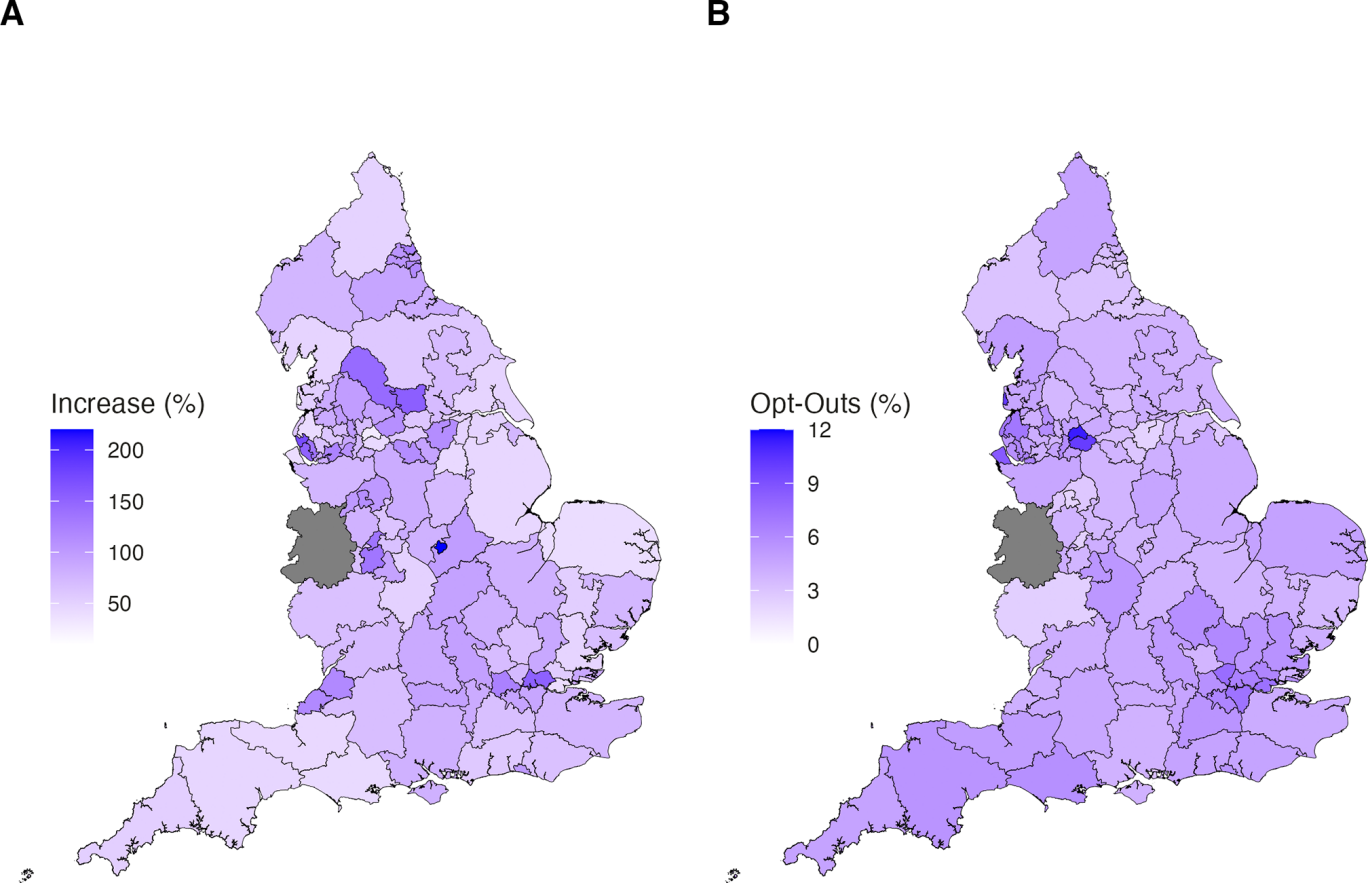
Trends in opt-outs by clinical commissioning group (CCG). (**A**) Increase in percentage of opt-outs by CCG between the end of May and June 2021. (**B**) Overall percentage opt-outs by CCG as of end of June 2021. The grey area represents missing opt-out data for NHS Shropshire, Telford and Wrekin CCG

Trends in opt-outs by CCG are presented in Figures 2 3 (data available in Supplementary Tables S1–S3). There were missing opt-out data for NHS Shropshire, Telford and Wrekin CCG.

**Figure 3.**
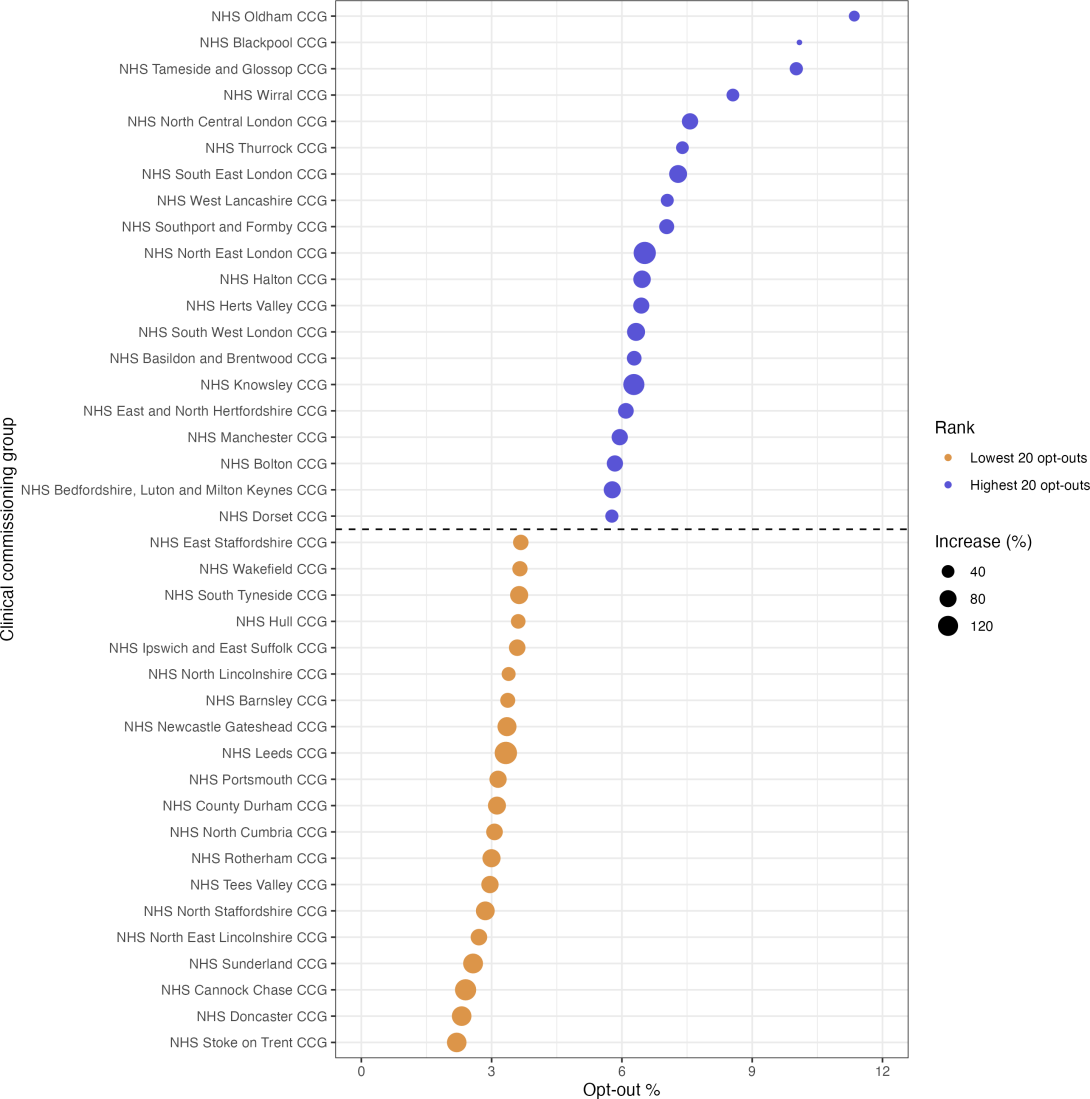
Clinical commissioning groups (CCGs) with the highest and lowest percentage of national data opt-outs at the end of June 2021 scaled by the increase from end of May 2021

Figure 2A shows increases in the percentage of opt-outs by CCG between the end of May and June 2021, ranging from 14.30% (NHS Blackpool CCG) to 219.96% (NHS Leicester City CCG). Figure 2B shows the overall percentage opt-outs by CCG as of the end of June 2021, ranging from 2.31% (NHS Doncaster CCG) to 11.35% (NHS Oldham CCG). Figure 3 presents the CCGs with the highest and lowest percentage of opt-outs as of end June 2021, with the increase in opt-out percentage denoted by the size of the points. There are no clear patterns in levels of opt-outs by CCG. However, Figure 2B highlights possible clustering around the Greater London and North West areas and Figure 3 suggests that smaller increases occurred in regions where opt-out levels were the highest.

**Figure 4.**
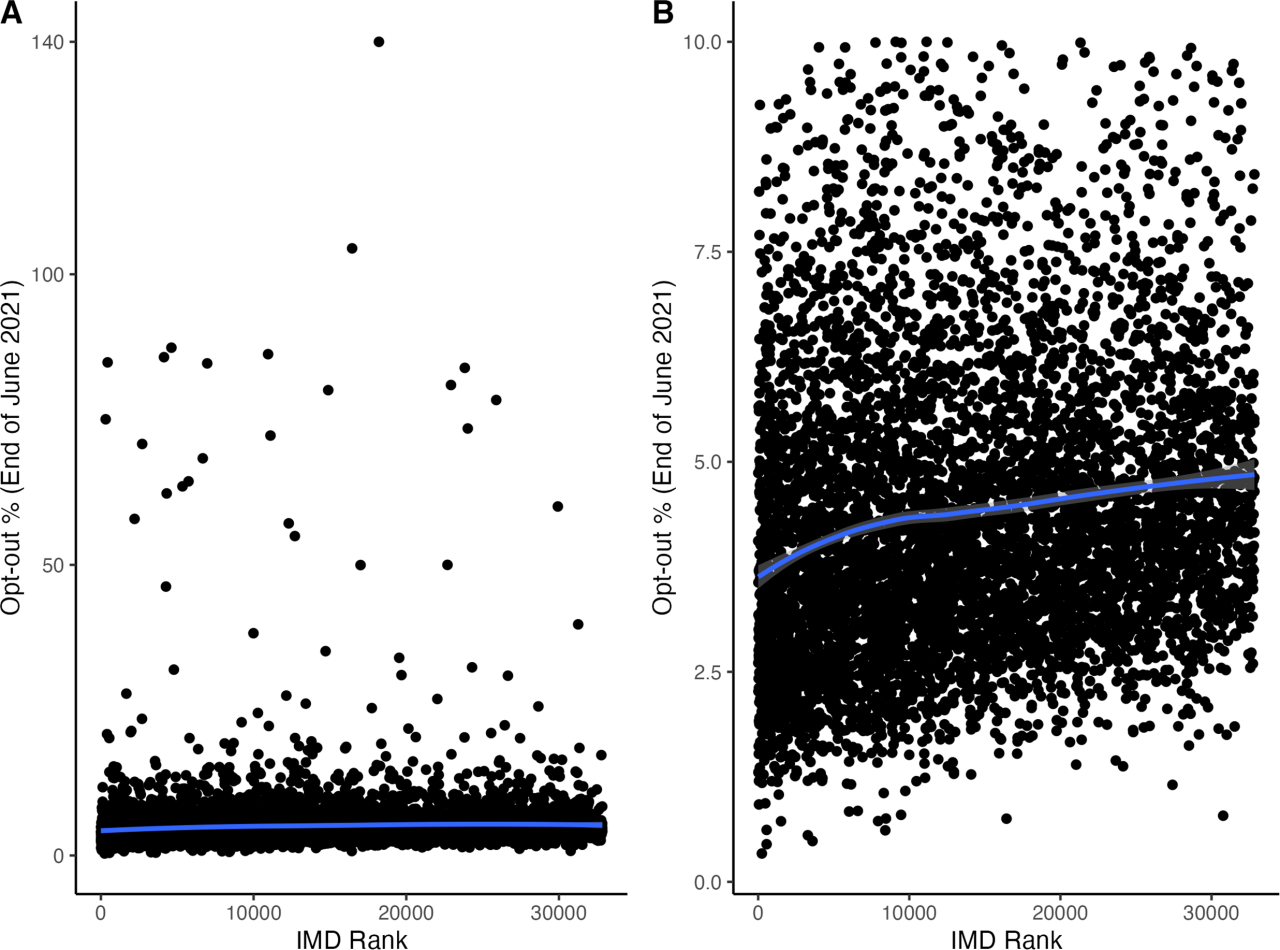
Comparison of total opt-out percentage by general practice at the end of June 2021 versus Index of Multiple Deprivation (IMD) 2019 rank with LOESS smoothed fit curve (General practices with available opt-out and IMD data: *n* = 6570; 99.7%). (A) Full data. (B) Restriction to opt-out per cent ≤10%. IMD rank orders areas from most deprived (that is, IMD rank = 1) to less deprived

Out of 6587 practices, 15 practices had missing opt-out or population-size data and a further two had missing IMD-rank data. Figure 4A demonstrates that while the vast majority of general practices have total opt-out <10%, there is no clear trend between those with the highest opt-out percentage and IMD rank. Across all levels of opt-outs, Figure 4 shows large variation and no clear trend in the relationship between general practice-level opt-outs and IMD rank.

### Impact of opt-outs on epidemiological analysis

Electronic health records (EHRs) are an important source of data for epidemiological research. They are large databases with rich, detailed information that can inform public health policy and clinical decision making. It is therefore important to understand the potential impact of opt-outs on research using EHRs. Here, we outline two major concerns if opt-outs become increasingly common: sample size, and bias.

#### Reduced sample size and power

Owing to their volume, EHR databases can be used to study rare diseases, interventions, and population subgroups. However, opt-outs reduce sample size and therefore power and precision of such studies. As an illustrative example, we consider a hypothetical study investigating the rate of a rare cancer. A historic study showed that the rate was previously 1 case per 100 000 person-years. Using large EHR data for 10 million adults for 1 year without opt-outs, researchers observed that the rate of this cancer is now 1.22 cases per 100 000 person-years. Supplementary Figure S2 shows that with this full dataset, the study had 80% power to detect this change in the rate of the cancer from 1 to 1.22 cases per 100 000 person-years (with 5% significance). However, if 5% of the study population opted out at random then the power would reduce to 78% and if the opt-out rate was 20% then the power of this test reduces to 71%. As the level of opt-outs increases, the probability of researchers having insufficient power to detect real differences (type II error) increases.^21^ Sample size reduction will be of particular concern for research of rare outcomes or in small subsets of the population, particularly if these groups are prone to opting out. For example, research into the association of learning disability with COVID-19 mortality was limited by the small overall number of events in children at the start of the COVID-19 pandemic,^22^ despite learning disability being strongly associated with mortality. Higher opt-outs among children (and potentially for children with learning disability) will lead to reduced sample size and limit the ability to draw meaningful conclusions and make timely policy recommendations.

#### Bias in observational studies of associations

**Figure 5.**
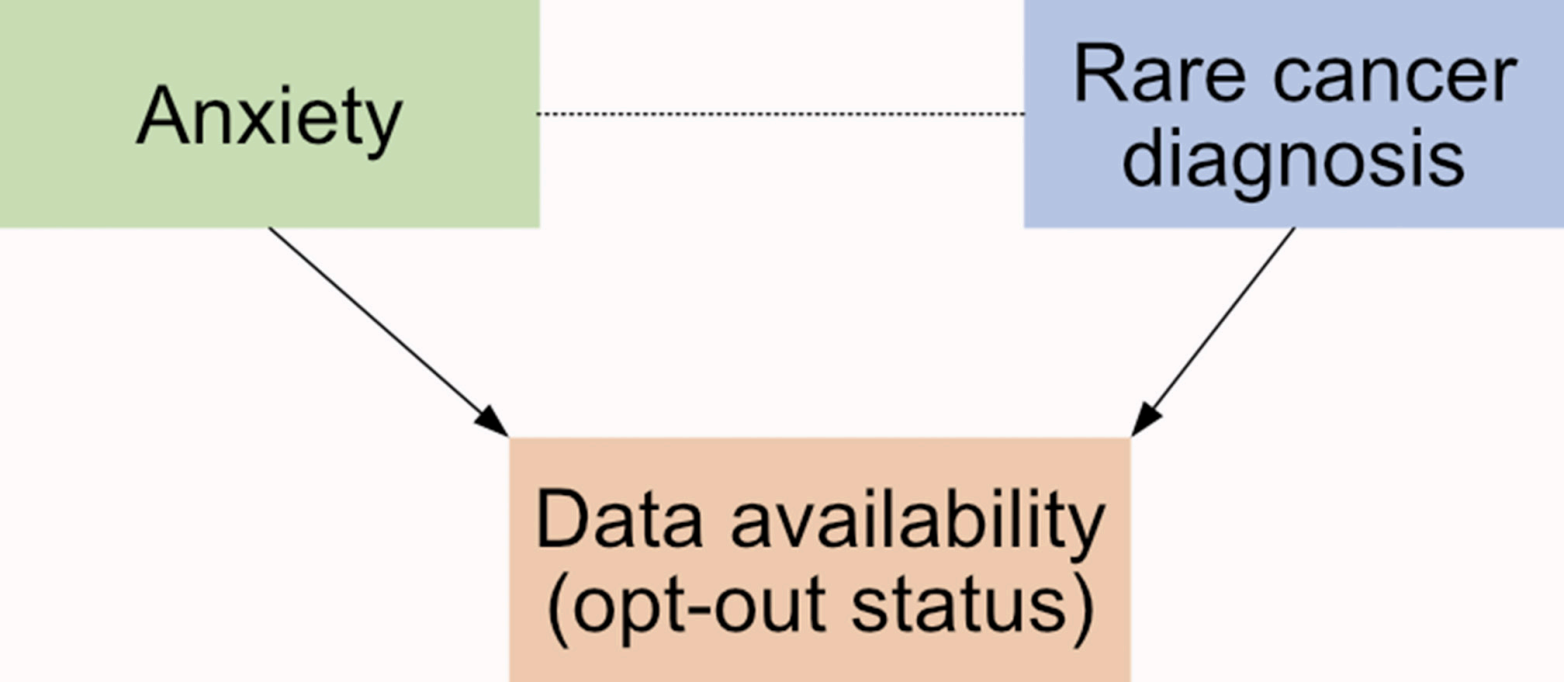
Illustrative example of how opt-outs could distort associations. We illustrate this using a hypothetical study investigating the causal effect of anxiety and diagnosis of a rare form of cancer in the general population. Research suggests that whether people have mental health experience is related to opt-out behaviour.^32^ For the purposes of this example, we also assume that whether a person has a rare cancer diagnosis is also related to their opt-out behaviour. If there is no true (causal) association between anxiety and this rare cancer diagnosis, analysing only people who have not opted out may induce an association in the data that is suggestive of an effect. Alternatively, if in truth anxiety slightly increases the risk of the rare cancer, analysing only people who have not opted out may mask or exaggerate this effect. Directed arrows indicate causal associations and dotted lines indicate induced associations

The second concern surrounds systematic differences between people who opt-out and those who remain in the data. Figure 5 illustrates the concern in a hypothetical study where having an anxiety diagnosis (the exposure in the study) or having a rare cancer diagnosis (the outcome) independently affects the individual’s likelihood of opting out. This could introduce a spurious (non-causal) association between anxiety and the rare cancer when none exists in truth, leading to needless concern among patients with anxiety about their cancer risk, wasted resources monitoring cancer risk in people with anxiety, and future research waste exploring a non-existent association. This can be considered similar to other methodological issues that have the potential to lead to the systematic exclusion of particular patient types such as missing data, linkage error, and collider bias.^23–26^ Supplementary Figure S3 illustrates the impact of these systematic opt-outs on study findings under different scenarios. In a study of 10 million individuals, we simulated 10% to have anxiety and 1% to have the rare cancer at random. Therefore, there is no association between anxiety and the rare cancer (odds ratio: 0.9996, 95% confidence interval [CI] = 0.98 to 1.02). If opting out is only associated with one of anxiety or rare cancer then the estimate is unbiased, but with less precision (Supplementary Figure S3). Estimates will be biased if individuals with anxiety or the rare cancer have a different propensity to opt-out compared with those without either health condition. Even at modest opt-out rates, if opting out is associated with either the exposure or the outcome (or a common cause of both), a spurious association may be observed and the study will be susceptible to demonstrating false positive associations (type I error).^21^

## Discussion

### Summary

Following the recent launch of the GPDPR initiative, total opt-outs almost doubled from 2.77% to 4.97% of the population registered with a general practice in England, with substantial variation by age, sex, and geography. At the end of June 2021, opt-outs were higher in females than males (5.53% versus 4.41%) and lowest in young children and the oldest adults. Across the period of interest, we observed the highest relative increase among people aged 40–49 years (2.89%–6.04%). Across CCGs, increases in opt-outs ranged from 14.30% (NHS Blackpool CCG) to 219.96% (NHS Leicester City CCG), leaving a reasonably consistent regional distribution over time, with potential clustering of key metropolitan areas in London and the North West. There was no consistent pattern between area-based deprivation measured by IMD rank and levels of opt-outs; although other practice-level factors, including practice size and primary care network membership, may have influenced the total proportion of opt-outs. Using a hypothetical study, we demonstrated a reduction in statistical power with increasing levels of opt-outs and distortion of findings where levels of opt-outs are associated with the study variables.

### Strengths and and limitations

Across a key consultation period in 2021, we have described the distribution of opt-outs in the population by age, sex, region, and area-based deprivation. We observed regional differences suggestive of higher levels of opt-outs in some cities and urban areas (for example, London, Liverpool, and Manchester); this could be driven by the system-level factors being associated with opt-outs. For example, opt-outs may be driven by key characteristics of these cities; relating both to people and contextual elements (for example, economical, political, and social environment).

The key limitation of our study is that opt-outs prevent the use of data for research. Therefore, while we had access to aggregated information describing opt-outs by age, sex, region, and general practice postcode-level deprivation data separately, stratified data describing intersections between these characteristics, patient-level data, and further information on clinical and sociodemographic characteristics of patients opting out is not available. This limited our ability to explore associations between a wider range of variables on opt-outs in the population and consider independent associations. This also prevented us from quantifying the magnitude of impact opt-outs are likely to have on a given research question. While we have described how characteristics associated with opt-outs vary over time and between geographical areas, we have not sought to describe the mechanism through which any increases have occurred. Theoretically, assessment through simulations is possible, but without more granular information it is not possible to generate data that are generalisable to specific research questions, other time periods, or opt-out systems in the UK and elsewhere. As a consequence, our exploration of the potential impact of opt-outs focused on a hypothetical example, specifically chosen to illustrate the key areas of concern. Additionally, while we present practice area-based deprivation data, this is only a proxy of individual socioeconomic status. Finally, while we present opt-out data until November 2021 (overall opt-out proportion 5.39%), as of January 2024, the overall proportion of opt-outs is 5.40% representing only an incremental change.

### Comparison with existing literature

This study updates research analysing UK opt-out data from 2017, before the launch of the current national data opt-out programme.^13^ Stark differences in opt-out proportions by geographic area were already present in 2017, following Care.data, with analyses suggesting that a small number of general practices were opting out patients by default rather than following requests from individual patients. Opt-out patterns by sex, age, and deprivation level appear similar to our observations from the end of May 2021, suggesting that the increased tendency to opt-out in younger adults by the end of June 2021 was directly related to the GPDPR initiative.^13^ At this time, descriptive analyses indicated that opt-outs were higher among Black people compared with White and Asian people; we were not able to repeat analyses by ethnicity as these data are not available.^13^ Studies based on Clinical Practice Research (CPRD) databases, which consist of de-identified data from a large proportion of UK practices and do not include people who have opted out,^27^ suggest that the database was broadly representative of the UK population by age, sex, area-based deprivation, and ethnicity in May 2021 and before.^28–30^ This suggests that the low proportions of opt-outs at this time, and small differences between demographic groups, had minimal impact on overall representativeness at this stage.

Survey-based research has indicated differences in willingness to share de-identified data for research without explicit consent by the demographic characteristics we identified and by the following characteristics that we were unable to study: ethnicity, education status, religion, and mental health. Being from a minority group correlates with higher concerns around sharing data, contributing to a paucity of research that is generalisable to those groups,^31^ whereas experiences of adverse mental health tends to be associated with being more likely to share data.^32^

Two major factors have been identified that impact an individual’s choice to opt-out: knowledge of who uses the data; and the potential public benefit of the research.^33^ This trust extends to researchers using the data, the healthcare system, and the government at large. Lack of trust may be mitigated by trust in science and demonstrable impact of research using these data. Similar to other research, our study shows that the interplay of these dynamics will vary across the population and therefore the pattern in opt-outs will be heterogeneous across a population.^31,32,34–37^

### Implications for research and practice

We have demonstrated that the number of people opting out of the use of their medical data for uses other than direct care varies by key demographic characteristics and increased following the launch of a recent government initiative. We have shown how increasing numbers of opt-outs may impact the feasibility of research and that resultant reductions in sample size and unmeasurable variation in opt-outs by a wider range of human characteristics may lead to unpredictable distortion of observed measures of frequency and association for individual studies. The level of this distortion will vary and be strongly related to the extent of opt-outs in the population of interest. Importantly, it is likely not possible to retrieve unbiased estimates of association; for example, by using quantitative bias methods, as data guiding the necessary assumptions is not available for people who have opted out.^38^ Without this information, a high proportion of opt-outs is likely to introduce unpredictable and intractable limitations to research, potentially giving rise to misleading results and suboptimal policy decisions.

It is vital that researchers work with other people to ensure that the impacts of opt-outs on research is included in key opt-out policy discussions, especially surrounding the planning and implementation of future national research databases and opt-out programmes. This is particularly relevant to ongoing discussions around the management of NHS federated analysis platforms by private companies, where recent YouGov data indicated this would likely result in a large increase in opt-outs.^39^

In conclusion, we have shown that while opt-outs reduce research quality, it is not possible to quantify or mitigate this impact adequately using data available to researchers. When planning future national research databases, policymakers should research the potential impact of proposed measures on the level of opt-out, how this varies between patient characteristics, and how it could be minimised.

## Data

The data used for analysis is provided at https://www.github.com/johntaz/nhs-opt-outs
